# *Quillaja saponaria* (Molina) Extracts Inhibits In Vitro *Piscirickettsia salmonis* Infections

**DOI:** 10.3390/ani10122286

**Published:** 2020-12-03

**Authors:** Hernán Cañon-Jones, Hernán Cortes, Mario Castillo-Ruiz, Trinidad Schlotterbeck, Ricardo San Martín

**Affiliations:** 1 Núcleo de Investigación Aplicada en Ciencias Veterinarias y Agronómicas, Facultad de Medicina Veterinaria y Agronomía, Universidad de Las Américas, Santiago 7500975, Chile; 2 Desert King Chile, Viña del Mar 2420505, Chile; hcortes@desertkingchile.cl; 3 Escuela de Química y Farmacia, Facultad de Medicina, Universidad Andres Bello, Santiago 8370146, Chile; mhcastilr@gmail.com; 4 Departamento de Ciencias Químicas y Biológicas, Facultad de Ciencias de la Salud, Universidad Bernardo O Higgins, Santiago 8370993, Chile; 5 Saponin Research Center, Santiago 7510132, Chile; t.schlotte.s@gmail.com (T.S.); rsanmartin@berkeley.edu (R.S.M.)

**Keywords:** plant medicines, SRS, fish pathology, antibiotic usage, salmonids, Chile

## Abstract

**Simple Summary:**

Bacterial diseases causes massive mortalities in aquaculture and antibiotic use remains the main measure to keep these under control. *Pisciricketssia salmonis*, an intracellular bacterium only present in Chile, produces high mortalities in farmed salmon and is currently the main reason for using antimicrobials compared to other salmon-producing countries such as Norway. Environmental and antimicrobial resistance concerns have been raised by the local and global public and society, although no scientific evidence has demonstrated such an impact. Thus, there is a constant search for new alternatives that can complement or reduce the use of antimicrobial in intensive salmon farming. Phytochemicals such as saponins from *Quillaja saponaria* extracts have been proven to prevent and control diseases in other animal production systems. This study explored the safety and efficacy of quillaja extract in in vitro infections with *P. salmonis*. The results of this study showed a good in vitro safety and efficacy to infections. The efficacy proved to be dependent on the quantity of saponins and toxicity dependent on purification. The results showed that quillaja extracts could be potentially used as a new sustainable and eco-friendly alternative to control *P. salmonis* infection, contributing to decreased fish mortality, antibiotic use and antimicrobial resistance in intensive aquaculture worldwide.

**Abstract:**

*P. salmonis* infections are the cause of major bacterial disease in salmonids in Chile, and the reason for using more antibiotics compared to other salmon-producing countries. Vaccination and antibiotics have not been efficient and new approaches are needed. The safety of *Quillaja saponaria* extracts was measured by cytotoxicity using flow cytometry of cytopathic and death of fish cell cultures and efficacy was assessed using in vitro infection models with pathogenic *P. salmonis*. Cytotoxicity was low and control of in vitro infections was achieved with all products, with protection of over 90%. Minimum inhibitory concentrations were much higher than those in the infection using cell cultures. These results suggest a dual mechanism of action where less purified extracts with a combination of saponin and non-saponin components simultaneously decrease *P. salmonis* infection while protecting cell lines, rather than exerting a direct antimicrobial effect. Quillaja saponins controlled in vitro infections with *P. salmonis* and could be considered good candidates for a new, safe and sustainable method of controlling fish bacterial infectious diseases.

## 1. Introduction

Global aquaculture production has steadily increased in the last two decades, achieving 114.5 million tons in 2018 [1], and Chile has become the second highest producer of salmonids in the world, with 924 thousand tons of salmon produced in 2018 [2]. Intensive animal production has historically been the subject of concern because they are associated with poor animal health and welfare [3]. Today, most animal production systems are managed sustainably [4], with continual incorporation of new technologies to improve the health and welfare status of animals, such as precision farming [5] and remote sensing [6]. However, a negative and biased perception of intensive animal farming persists in consumers and the general public [3]. This also has been the case for aquaculture, where the main concerns have been disease outbreaks related to high stocking densities [7], excessive use of antimicrobials leading to bacterial resistance [8] and negative environmental impacts of fish farming such as degrading seafloor contamination [9] or invasion of salmon escapees to the sea, rivers and lakes [10].

Outbreaks of disease remains a major problem in animal production [11,12], including aquaculture [13]. Particularly, bacterial and viral diseases have been identified for salmonids with direct negative effects on health, welfare and production worldwide [14]. Undoubtedly, the most important salmon bacterial disease currently in Chile is Septicaemic Rickettsial Syndrome (SRS) accounting for 54.5% of mortalities associated with infectious diseases during the seawater growing phase [15]. The disease is the main reason for the use of antimicrobial therapy in the Chilean salmon industry [16]. *Piscirickettsia salmonis* is the causative agent of SRS, a Gram-negative bacterium infecting multiple internal organs [17]. The bacteria is a facultative intracellular pathogen [18] invading specific and crucial immune cells such as monocytes and macrophages [19,20,21]. Presentation of the disease can be acute or chronic [18]. High mortality of up to 90% of all animals can occur during acute infections [22]. The sublethal and chronic presentation usually occurs with clinical signs including non-specific signs such as darkening of the skin, slow surface swimming and inappetence [22]. Typical external signs of the diseases have included pale gills, haemorrhages in the base of fins and skin ulcers. Internally, the main and pathognomonic sign is the presence of a small abscess in the liver [17]. A presumptive diagnosis is carried out, taking into account clinical and necropsy signs, and conformation is made by laboratory analysis including ELISA, IFAT and PCR [18].

Preventive measures such as vaccines have been developed with little or no proven on-farm efficacy [23], because a mainly humoral response is achieved instead of a cellular mediated, which is needed in the case of an intracellular bacteria such as *P. salmonis* [24]. The evidence from salmon producers on the effectiveness of vaccines for SRS prevention under field conditions, including the use of the live vaccine introduced in the market, also suggests that long-term protection is variable and limited. Freshwater vaccination strategies have a better immune response in some fish species, and the variation in susceptibility to SRS outbreaks may be influenced by genetic differences and environment factors [24]. Genetic selection has also been explored, with relatively low or unknown success and application [25]. In part, this can be explained because genetic resistance to SRS is a polygenic trait, with more than 100 candidate genes explaining the resistance in Coho salmon, Atlantic salmon and rainbow trout in Chile [26].

The most common control measure for SRS outbreaks is the use of antibiotics given in the feedstuff. Fish infected with *P. salmonis* must be treated with an antibiotic, as it is unethical and illegal to maintain sick or morbid animals by current Chilean law, protecting the health and welfare of farm animals, including fish [27,28]. Antibiotic treatment for SRS consists of prolonged or repeated treatments due to the intracellular avoidance strategies of the pathogen [29]. The use of antibiotic treatment for SRS has been the subject of discussion amongst several non-governmental institutions and the non-scientific community, claiming potential but non-evidence-based environmental and human health effects [30,31,32]. This has led to an unsubstantiated and unjustified negative stigmatization of the salmon industry in Chile and worldwide [33,34]. Antibiotic use in the Chilean salmon industry has been constantly decreasing in the last few years, from 563 to 322 tons between 2014 and 2018, due to the implementation of a set of coordinated efforts from the public sector and salmon industry, such as the Active Surveillance Program for SRS by the for National Fisheries and Aquaculture Service of Chile [35] and the implementation of the *Good Practices for Antimicrobial Use in Aquaculture* handbook [36].

The therapeutic arsenal for the treatment of bacterial diseases in aquaculture in Chile is limited to only a few drugs, such as oxolinic acid, flumequine, erythromycin, amoxicillin, doxycycline, florfenicol and oxytetracycline [37]. Although oxolinic acid and flumequine are registered for use in chilean aquaculture, they are not currently used in food-producing animals following the recommendation of exclusive human use by the World Health Organization, and erythromycin, amoxicillin and doxycycline are mainly used for breeding animals [36]. Thus, florfenicol and oxytetracycline are the main drugs used for SRS in Chile.

With the limited amount of antimicrobials registered for use in Chilean salmon industry [38], several bioactive and natural products have been developed to help in preventing and controlling salmon diseases such as probiotics, immunostimulants and phytochemicals and other plant extracts, which can promote fish health and welfare [39]. Saponins have been used in numerous applications such as antibacterial [40,41], antiviral [42], antifungal [43] and antiparasitic [44]. The proposed mechanism of action of saponins is based on their chemical interactions with lipids of biological membranes or layers, particularly with cholesterol, leading to alterations in membrane integrity [45], blocking the adhesion of viruses and bacteria [46] or permeabilising membranes [47]. There are various sources of saponins from animal [48,49], plants [50] or synthetic [51,52]. Quillay (*Quillaja saponaria* Molina) is a native tree of Chile containing a high concentration of saponins that are industrially obtained as a powder or liquid extract [53]. The main uses of quillaja saponin extracts are as emulsifiers in cosmetics, food and beverages and as vaccine adjuvants [54]. They have also been commercially used as a biocide to invertebrates such as nematodes (US 20050074508) and molluscs (US 20070196517) or fungi [43]. However, no studies have reported the use of quillaja saponins on bacterial infections in fish species, except those described by authors’ patents (described in the Patents section, below), where *Quillaja saponaria* extracts were used in feedstuff in salmonids.

The aim of this work was to determine the in vitro effect of quillaja saponins extracts on *P. salmonis* infection.

## 2. Materials and Methods

### 2.1. Quillaja Saponaria Products and Preparation

QuillajaDry^®^ 100 (QD100, powder extract, mainly containing triterpene saponins ca. 25% *w/w*), UltraDry^®^ 100-Q (UD100-Q, powder extract, mainly containing triterpene saponins ca. 65% *w/w*) and VaxSap^®^ (VS, highly purified powder extract, mainly containing triterpene saponins ca. 90% *w/w*) were used and obtained from Desert King Chile S.A., Chile. It is important to note that Desert King S.A. also has liquid extracts available, but the selection of the quillaja extracts as powders was made because ingredients [55] and additives in fish feeds [56] are normally incorporated in powder form during salmon feedstuff production. An initial stock solution of 1 mg/mL was prepared for each product by dilution with non-supplemented L-15 medium at 37 °C for 3 h under gentle stirring, and then filtered through a nitrocellulose membrane 0.22 μm and refrigerated (8 °C) until further use. The dilutions used for the evaluation of in vitro safety and efficacy were prepared using serial dilutions ranging from 0 to 500 μg/mL.

### 2.2. In Vitro Safety/Cytotoxicity Assay of Quillaja Saponins Products in Salmon Cell Lines.

SHK-1 cell line was used for cytotoxicity assays. SHK-1 cell line (*Salmo salar*; ECACC 97111106 Number, European Collection of Cell Culture, Salisbury, Wilts SP4 0JG, UK) was cultured at 15 °C in L-15 medium supplemented with 10% *v/v* foetal bovine serum, 4 mM L-glutamine, 1% *v/v* 2-mercaptoethanol and 50 μg/mL gentamicin.

Incubation of 5 × 10^5^ cells/well in 2 mL of culture medium for 72 h at 15 °C was carried out, then the culture medium was replaced with fresh medium and cell confluency was checked. If 100% cell confluence was achieved, a further 24 h incubation period was carried out to allow setting the cell culture. Then, culture medium was replaced with the different quillaja extracts for 24 h for cytotoxicity test. After 24 h incubation, cells were washed twice with cold PBS (ca. 8 °C) and then disrupted using a solution with 0.05% trypsin and 0.02% EDTA. Cells were analysed by flow cytometry FACS Canto II (Becton Dickinson^®^, Franklin Lakes, NJ, USA) and cytosol incorporation of propidium iodide (PI) method [57] was determined as a marker for dead cells. Cells incubated with a solution of ethanol 100% were used as positive control, while cells incubated without quillaja extracts subjected to the same conditions were used as negative control. A probit regression model (Finney, 2009) was used to obtain the cytotoxicity concentration (CC_50_, 50% toxicity; CC_90_, 90% toxicity).

### 2.3. Quantification of P. Salmonis Using Quantitative Polymerase Chain Reaction (qPCR)

Briefly, the gene encoding for 16S rRNA (Fw: 5′-AGG-GAG-ACT-GCC-GGT-GAT-A-3′; Rv: 5′-ACT-ACG-AGG-CGC-TTT-CTC-A-3′) was amplified as described [19]. Genomic DNA was obtained using the Wizard™ Genomic DNA Purification kit and PCR amplification was performed using PowerUp™ SYBR^®^ Green master Mix (Thermo Scientific, Waltham, MA, USA). Primers were added to a final concentration of 0.4 μM, and 12 ng of template was used. The qPCR was carried out on a QuantStudio 3 Real-Time PCR system (Thermo Scientific) and the quantification of 16S rDNA copies was calculated by interpolation from the standard curve with the cycle threshold (Ct) value obtained for each sample [19]. The results are expressed as 16S rDNA copy/cell.

### 2.4. In Vitro Efficacy of Quillaja Saponin Products Against P. Salmonis Infection on CHSE-214

CHSE-214 cell lines were used for efficacy in vitro studies. CHSE-214 cell line (*Oncorhynchus tshawytscha*, 91041114-1VL, Merck) was cultured to monolayers and incubated at 2 × 10^6^ cells/well until reaching a confluence of >70%, using Eagle’s minimum essential medium supplemented with 10% foetal bovine serum, penicillin (100 IU/mL) and streptomycin (100 μg/mL). Then, the culture medium was removed and the monolayer was infected with a bacterial suspension containing 10^5^ genome copies/mL of culture medium of a highly pathogenic Chilean *P. salmonis* (LF-89 wild-type isolate, donated by ActivaQ S.A.), at the same time as quillaja extracts concentrations prepared from the stock solution, as described previously. The concentration of quillaja extracts used was at least 10-fold lower than CC_50_ estimated in the cytotoxicity assays. After 24 h infection, *P. salmonis* RNA was quantified by qPCR and compared with the control (no *P. salmonis* added). The percentage of inhibition was expressed as the relative percentage between treatments and control. All experiments were carried out in triplicate.

### 2.5. Minimal Inhibitory Concentration (MIC) on P. Salmonis

Tests for quillaja saponin susceptibility were prepared according to the instructions given by the CLSI, guide M49-A (CLSI 2006), but introducing the ADL-PSB medium for *P. salmonis* growth [58]. Stock solutions of Vax Sap were prepared with sterile distilled water. Ninety-six well microplates containing dilutions from 0.0027 to 23.000 μg/mL of VaxSap (0.0024–20.700 μg/mL of saponins) were inoculated with 5.0 × 10^5^ CFU well−1 of *P. salmonis*. Microplates were incubated statically at 19 ± 2 °C for 5 to 7 days, as this is a non-cell medium, in accordance with previously described methods [58]. Absorbance was measured at 580 nm for each well. All experiments were performed in triplicate.

### 2.6. Statistical Analysis.

Descriptive statistical analysis was carried out for each product according to evaluation time and concentrations used. Infection rates of *P. salmonis* trials were calculated by comparing DNA quantification of quillaja treated and untreated controls. Statistical differences in the concentration and time for each quillaja product were measured using analysis of variance and multivariate analysis of variance. All statistical analyses were carried out using the R (R Development Core Team, 2014). A probit regression model (Finney, 2009) was used to obtain the cytotoxicity concentrations (CC_50_, 50% cytotoxicity; CC_90_, 90% cytotoxicity).

## 3. Results

**Figure 1:**
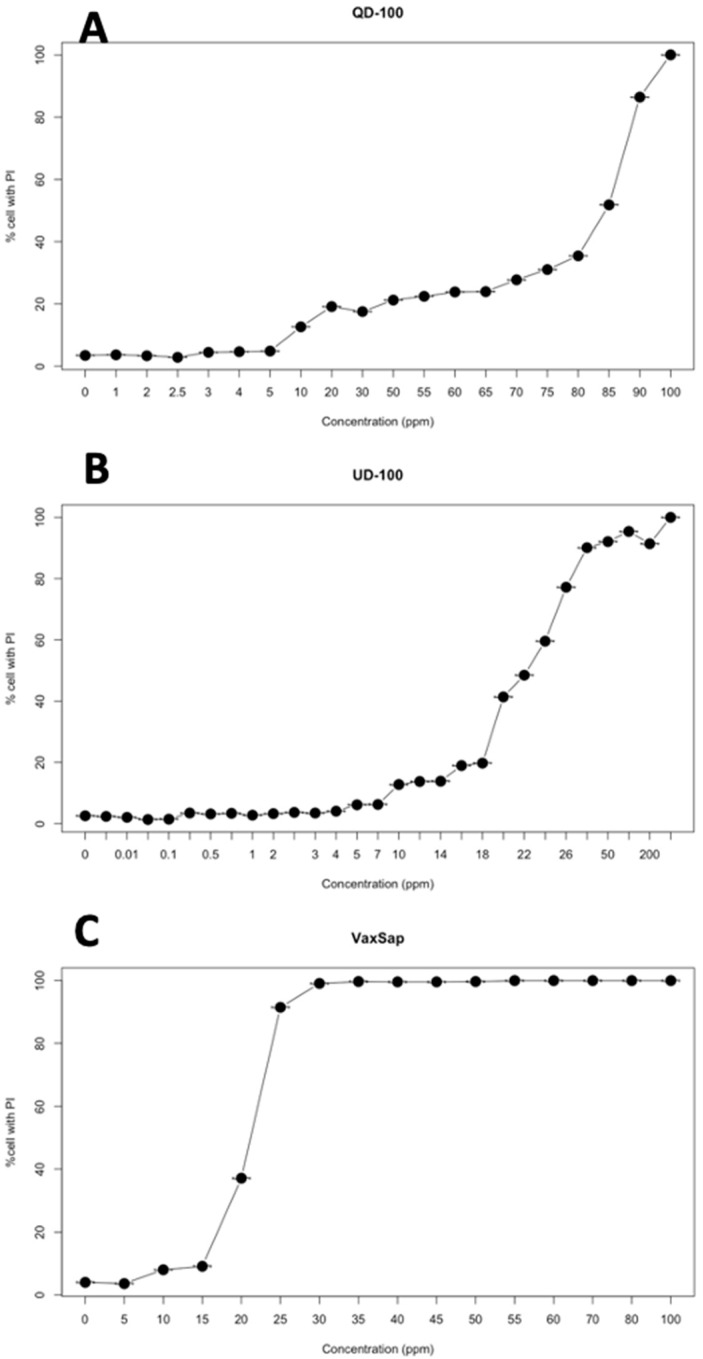
Cytotoxicity curves of quillaja extracts on SHK-1 cell culture: (**A**) QD 100; (**B**) UD100-Q; (**C**) VaxSap.

Quillaja extracts had low toxicity on SKH-1 cell cultures with a CC_50_ between 20.4 and 83.4 μg/mL and a CC_90_ between 25.3 and 92.6 μg/mL, depending on the product used (Figure 1, Table 1). However, the toxicity expressed in terms of saponin concentration was lower, with 14.4 to 20.8 μg/mL for CC_50_ and 19.0 to 23.2 μg/mL for CC_90_ (Table 1).

All quillaja products effectively inhibited bacterial infection of *P. salmonis* in vitro, ranging from 37.13% to 99.99% (Table 2). This range is wide and it is explained by the purification differences in the products tested.

**Figure 2:**
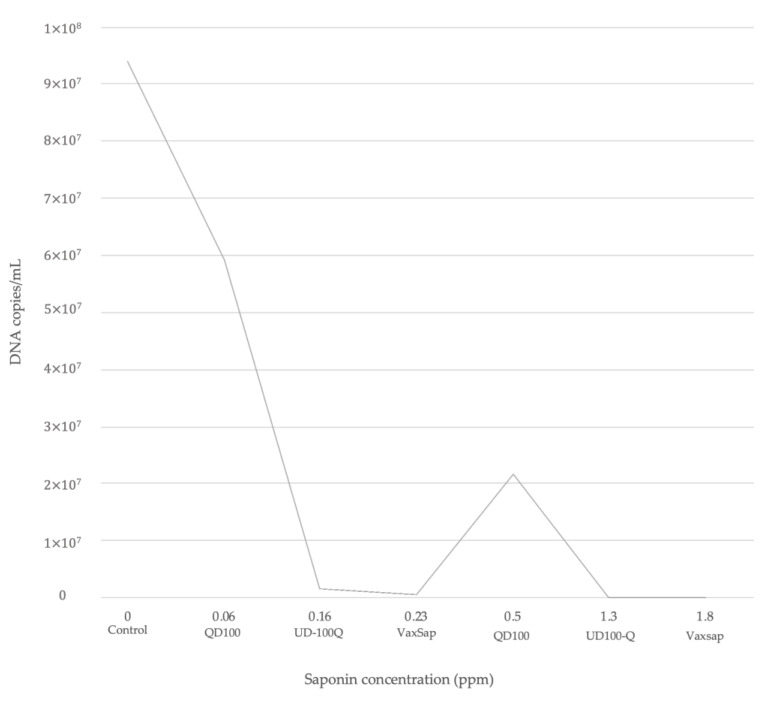
Reduction of *P. salmonis* (Mean DNA concentration) according to saponin content for each quillaja product.

Saponins rich products such as Vax Sap and UD100-Q had a potential of reduction in infection by 10- and 1000-fold, respectively (Table 2). The level of reduction fold was saponin concentration-dependent (Figure 2), with lower *P. salmonis* DNA concentrations post-infection when the cells were treated with a high concentration of saponins (Table 2, Figure 2). However, QD100 product showed a lower protective effect in comparison with UD100-Q and VaxSap (37.13% vs. 98.29% vs. 99.48%, respectively). Cells pre-treated with 0.5 ppm of saponins from QD100 showed a DNA concentration of *P. salmonis* to 2.2 × 10^7^ DNA copies/mL, very similar to the control group. On the other hand, when cells were pre-treated with 0.23 ppm of saponins from Vax Sap, DNA concentration of *P. salmonis* was reduced to 2.4 × 10^3^ DNA copies/mL (Table 2, Figure 2), These results suggest that saponin concentration and extract purification could be involved in the observed effect.

The direct minimal inhibitory concentration (MIC) was very high (11,500 μg/mL of product or 10,350 μg/mL of saponins), which was approximately 5000-fold higher than the doses of saponin used in the infection assays.

## 4. Discussion

Extracts from *Quillaja saponaria* had a protective effect against *P. salmonis* in an in vitro cell infection challenge (23 to 99% protection post-infection) and is the first evidence of quillaja saponins controlling bacterial infections in fish cell lines. The doses which affected over 90% of the infections were low (up to 1.8 μg/mL), which suggest that extracts could readily be included in fish feed for treatment of infections during salmon production. Our study also showed a direct relationship between the reduction in bacterial infection with the saponin concentration. Quillaja products containing more than 65% of saponin (UD-100Q or VaxSap) significantly reduced bacterial infection, while Quillaja extracts with saponin concentrations between 20% and 25% (QD-100) had a comparatively higher infection rate than other products.

The replacement of antimicrobials used in aquaculture for more sustainable and natural alternatives has been the subject of recent interest worldwide by the WHO and FAO [59]. The main reason for looking at alternatives to antibiotics is the potential of antimicrobial resistance in both animal production systems [60,61]. Phytochemicals such as saponins are good candidates to be used and tested against infections in animals and humans [62,63] and, with the results of this study, future research should be conducted to assess the efficacy of extract treatment under natural conditions and any residues in products derived from fish. Using classical non-cell culture techniques such as MIC, the values found in this study were higher than those observed against Gram (−) bacteria *Salmonella Typhimurium* and *Escherichia coli* or Gram (+) bacteria *Staphylococcus aureus* [64], suggesting that fish pathogens are less susceptible to direct action of saponins and a different mechanism of action against pathogen infection may be involved. This may be sustained because saponins have been proven to modify the permeability and fluidity of host cell membranes [45,65,66], which may affect the attachment of pathogens. In vitro and in vivo studies suggest that quillaja saponins “cover” host cells, preventing the contact of different types of viruses to their binding sites [67] and by changing protein–protein interactions, which may reduce colonization in cells by the pathogen [68]. This is of particular importance, because we added the saponin extract alongside the bacterium inoculum, at saponin concentrations far below any MIC effect (pathogen), suggesting a protective effect directly on the host cells’ membrane.

These effects have been shown in vitro and in vivo for viral infection with rotavirus, herpesvirus and HIV [42,46,67]. In bacteria, this is supported by a study conducted by Arabski et al., 2009 [69], which showed that the use of saponins decreases the quantity of antibiotics which are needed to eliminate *Proteus mirabilis*, showing a potential effect on host membranes. Quillaja extracts contain a non-saponin fraction consisting mainly of phenols and some others in lower concentrations, which may play a role in the protective effects against *P. salmonis*. The phenolic components in quillaja extracts exert antioxidants agents such as piscidic acid, representing 75% to 87% of the total level of phenols, and derivatives of *p*-coumaric acid, representing 8% to 20% of phenol totals [54]. The concentration of phenols in QD-100 is higher than those present in VaxSap or UD100-Q, suggesting that a higher concentration of phenols may exert an effect by protecting the survival of *P. salmonis* via antioxidative response during the infection cycle of the pathogen. Future studies should be conducted to clarify the exact mechanism of action of phenols and saponins against infection with *P. salmonis*, and saponin residues in muscle and edible organs studies should also be conducted. From an economic point of view, producing less purified quillaja extracts that show good safety for fish and efficacy in controlling *P. salmonis*, such as QD-100 may prove more technically and economically feasible than highly purified extracts for quillaja producers for the salmon industry.

The toxic effect of quillaja extracts on fish culture cell lines used in this study was higher than the dose used to inhibit infection with *P. salmonis* (14.4–20.8 vs. 0.16–1.8 μg/mL), which suggests good safety margins for future in vivo studies. Products with more purification were more toxic than those that were less purified (VaxSap and UD-100Q vs. QD-100), suggesting that the non-saponin fraction of the extracts (consisting mainly polyphenols) may have a protective effect on fish cell lines. Comparatively, the CC_50_ obtained for the products were lower than those reported in other mammal culture cell lines such as L929, MA-104, Vero, BS-C-1 and CEMx174 [42,67]. One explanation for this result is that the fish culture cell lines used in our study may be more sensitive to the action of saponins, an issue to look into in future in vitro studies using fish cells lines. The toxicity in the fish cell line used here was very similar to those observed in vitro, conducted using quillaja saponin extracts on human gastric cancer cells lines SNU1 and KATO III [70], and human maxillary sinus squamous cancer cell lines HNSCC [71] showed similar toxicity results (13 to 67 μg/mL). These results suggest that cancer cell lines may have changed the structure of the cell membrane, as supported by previous studies [72]. The discovery presented here may be further explored in the future using similar cell membrane stability studies [45].

## 5. Patents

WO2018018170A1-Use of extracts of *Quillaja saponaria* for the prevention and control of bacterial infections in fish—Google Patents Available online: https://patents.google.com/patent/WO2018018170A1/en (accessed on 10 August 2020).

US20200023026A1-Method for preventing and controlling bacterial infections in salmonid fish using *Quillaja saponaria* extracts—Google Patents Available online: https://patents.google.com/patent/US20200023026A1/en (accessed on 10 August 2020).

## Figures and Tables

**Table 1:** Cytotoxicity (CC_50_ and CC_90_) of quillaja extracts on SHK-1 cell culture.

Quillaja Extract	CC_50_ (µg/mL)		CC_90_ (µg/mL)	
	Product	Saponin	Product	Saponin
QD 100	83.4	20.8	92.6	23.2
UD100-Q	22.1	14.4	29.2	19.0
VaxSap	20.4	18.4	25.3	22.8

**Table 2:** In vitro efficacy of quillaja extracts in cells against *P. salmonis.*

Treatment	Product Concentration (mg/mL)	Saponin Concentration (μg/mL)	Ct (dRn)	DNA (Copies/mL)	Inhibition (%)
Control	0.00	0.00	14.72	9.4 × 10^7^	0.00
QD100	2.00	0.50	14.87	2.2 × 10^7^	76.94
QD100	0.25	0.06	14.73	5.9 × 10^7^	37.13
UD100-Q	2.00	1.30	24.37	3.4 × 10^4^	99.96
UD100-Q	0.25	0.16	20.07	1.6 × 10^6^	98.29
VaxSap	2.00	1.80	27.98	2.4 × 10^3^	99.99
VaxSap	0.25	0.23	20.98	4.9 × 10^5^	99.48
